# Chicago Dental Society

**Published:** 1884-09

**Authors:** A. E. Baldwin


### CHICAGO DENTAL SOCIETY.

### JULY MEETING.

REPORTED FOR THE INDEPENDENT PRACTITIONER BY A. E. BALDWIN, M. D.

The paper of the evening was by Dr. A. W. Harlan; the subject, "Gold and Tin Combined as a Filling Material."

The essayist said that attention to present dental literature develops the fact that the tendency of to-day is to deal mostly with histological conditions, causes and treatment. This being the fact, he had ventured to present his paper on the above subject, as we all must admit that the filling of teeth (for the future as the past) will occupy the greater part of the dentist's time. He would state it as his belief that gold is the best material for filling the greater proportion of tooth cavities; but in some cases it is not practicable:

First, because of inability of the patient to pay its price. Second, lack of endurance for protracted operations on the part of the patient. Third, the impossibility of making them uniformly good for young, aged and weak. Fourth, because of the habits, age, or condition of the patient. Fifth, in the majority of children under sixteen to eighteen years of age. Sixth, the certainty of failure in certain teeth at any age. And in addition, every thinking dentist will find that in certain cases other material is better than gold.

We have amalgam, tin, Robinson's fibrous material, gutta-percha, oxy-chloride and oxy-phosphate of zinc, and other combinations, among which is gold and tin. With amalgam he would advise that it be used only in obscure places, or in crowns of lower molars. Condemns its use underneath large gold fillings, when other materials may be used more advantageously, and serve a better purpose.

Tin may be used in the first part of proximal fillings in molars or bicuspids, and in any temporary teeth. It may be used in small crown cavities, in children's permanent molars, and in third molar cavities; also in many cases in other teeth where the cavity is not in view. Gutta percha, as a rule, should be employed only in temporary teeth. Oxy-chloride of zinc should only be used in pulpless teeth, and then be covered with a metallic filling. Oxyphosphate of zinc may be used in teeth with or without pulps, but should in every case be covered with metal. In proximate cavities in incisors he would prefer oxy-phosphate to the oxy-chloride of zinc, because it is less irritating.

The combination of which this paper is written originated with Dr. F. P. Abbot, of Berlin, in whose hands it has successfully stood the test for more than thirty years. He does not use it simply to save gold, but finds that certain teeth can be saved with this combination that could be saved with neither gold, tin, nor amalgam. He prepares the material by taking a sheet of about No. 4 tin foil, and laying on it a sheet of No. 4 non- or semi-cohesive gold foil, folding them together so as to have the tin inside the gold, or twisting them in a roll, the gold outside, and (producing a set of six instruments) with these instruments he can fill any cavity. The instruments are modified forms of fine soft gold pluggers. Since adopting the above combination the essayist has almost abandoned amalgam. He commended the gold and tin for its density, easy insertion, capacity for fine finish, its non-conducting and non-shrinking qualities, and its compatibility with tooth structure. The chlorides and oxides of tin are also antiseptic. Those who have not used this combination will be surprised at the rapidity with which it may be used. It may be employed in any cavity not exposed to view, and in buccal, crown and proximal cavities in molars and bicuspids. The cavity should be prepared so as to be retentive in shape. He has used it largely in proximal cavities extending over the crown, and finds them resisting wear to an astonishing degree. It may be used anywhere that amalgam can, and with more certainty of non-leakage, and has the additional advantage that it can be finished at the same sitting. In the manipulation of this combination care is necessary to avoid chopping up the material in packing it. In answer to a question the essayist said he would not use a mallet until the filling was all in.

DISCUSSION.

*Dr Allport*—Premised his remarks by saying that this, though an old and hackneyed subject, was one of prime importance, and we could not know or hear too much of it. No one could understand

*too* well how to fill teeth. Fillings exert their influence in saving the teeth in two ways; one by hermetically sealing, and the other by the therapeutical effect of the filling material. The method spoken of in the paper was not new, and the instruments used are almost the same as those used for soft gold. He believes the most important power exerted is in the therapeutical effect, as tin when brought in contact with the fluids of the mouth is acted upon, and produces oxide of tin. We get also a sulphate, and by a chemical interchange the sulphide. It is well known that the oxide of tin is destructive to all the low forms of animal life. But with the material used as spoken of in the paper, the tin is excluded from the contact with fluids, being covered with the gold. Hence we would not get the therapeutical effect.

*Dr. Cushing*—Said he was rather skeptical as to the therapeutical effect of any material; he believes the tin has attained its notoriety over other filling materials more by its adaptability than from any therapeutical reason.

*Dr. Marshall*—Said he had seen fillings which had been put in by Dr. Wescott, of Syracuse, New York, where tin had been used in the bottom of the cavity. In these cases he found the tin crystallized. The tooth discolored some, but in appearance it was perfectly preserved under the tin. He is a firm believer in the therapeutical action of fillings, especially of tin. He spoke of Dr. Kingsley's method of patching gold fillings with tin or amalgam, and as proof of the preservative effects of tin cited the burial cases in England, which were tin-lined. The parts of wood in contact with the tin lining, and the copper nails, were preserved when other portions had long since decayed. In a live tooth moisture is present in all cases in tooth structure, resulting in the natural formation, when tin is present, of the oxide.

*Dr. Hughes*—Cited a case similar to those spoken of by Dr. Marshall, where a filling had been in over thirty years, and after the filling was removed he could clearly distinguish the portion occupied by the tin, because of the absence of decay.

*Dr. Crouse*—Said he had never been a believer in the therapeutical action of fillings. He could endorse the remarks made by Dr.

Cushing. As a matter of fact, there were lamentably few perfect fillings, and in the defectiveness of the work may be found the cause of a large share of the failures of fillings. He does not see any advantages in Dr. Harlan's method of using tin and gold. He uses tin largely, but does not finish filling with it, because he wants something harder. If he gets perfect manipulation, and the co-operation of the patient (by keeping teeth cleansed), he has little trouble with failures. He thinks a reason why tin does as well as it does is its almost perfect adaptability. He uses tin pellets in difficult places, then finishes up with gold. Believes more pains should be taken to teach students how to manipulate soft or non-cohesive gold. He quoted cases where he had put in tin fillings in a hasty and imperfect manner, not expecting them to stand, and they had surprised him by perfectly preserving the teeth.

*Dr. Allport*—Said the cases quoted by Dr. Crouse would prove just the opposite of his statement; that is, they must have acted by therapeutical effect. Even accepting the correctness of the views of Dr. Crouse, tin would be the best material to employ, because it could be successfully used with much less skill. He thinks this method is advantageous in making solid and easily adapted fillings. No one can question the fact that the salts of tin are very destructive to the low forms of animal life; neither can there be any question but that bacteria produce decay.

*Dr. Koch*—Thinks the success of these fillings lies in their adaptability, not in the cohesiveness of the gold, nor in the therapeutical effect of the tin.

*Dr. Baker*—Firmly believes in the therapeutical effect of tin; does not believe in the wholesale condemnation of cohesive gold fillings; believes the trouble with them is almost wholly in faulty manipulation. Should advise the use of the ribbon form for fillings.

*Dr. Harlan*—Says he folds the tin and gold sometimes with the gold outside, and sometimes the tin; he uses it principally when he would otherwise use amalgam. It can be inserted very quickly. He does not believe in the therapeutical action of tin. Oxide of tin is a white, insoluble powder, and the sulphide of tin is not formed in perfectly filled cavities.

The Society adjourned to meet the first Tuesday in October, when Dr. Marshall will present a paper on "Pregnancy, and its effect upon the teeth of the mother and child"

